# Coxsackievirus Infection and Associated Diseases

**DOI:** 10.3390/microorganisms10081566

**Published:** 2022-08-04

**Authors:** Magloire Pandoua Nekoua, Didier Hober

**Affiliations:** Laboratoire de Virologie ULR3610, Université Lille et CHU Lille, 59000 Lille, France

Coxsackieviruses (CV) are ubiquitous and widespread single-stranded RNA viruses belonging to the *Picornaviridae* family and the genus *Enterovirus*, which also includes poliovirus (PV), the best known of the enteroviruses (EV). They are mainly transmitted by the fecal–oral route and are a major cause of viral infection each year worldwide, especially in children. They were first isolated in the late 1940s from the feces of paralyzed children living in Coxsackie (New York, USA) and were later classified into CV groups A (CVA) and B (CVB) based on the nature of their pathogenicity in mice. CV infections are considered to be asymptomatic or to induce subclinical or mild symptoms; nevertheless, they are also associated with various acute and chronic pathologies. For example, outbreaks of hand, foot, and mouth disease are mainly caused by enterovirus A71 and CVA, whereas CVB are often associated with the pathogenesis of chronic myocarditis, dilated cardiomyopathy, and autoimmune diseases such as type 1 diabetes (T1D). The knowledge of the cellular and molecular mechanisms of CV pathogenesis in human disease has progressed considerably in recent years, although questions still remain. This Special Issue presents the latest findings on CV virulence, mechanisms involved in the pathogenesis of CV-associated diseases, and therapeutic strategies to combat CV infections.

EV, including CV, initially replicate in the gastrointestinal tract or in the upper respiratory tract before spreading to various target organs (heart, pancreas, thymus, etc.) through the lymphatic and blood systems. It has long been thought that the primary replication of all EV takes place in Peyer’s patches (PPs) (a group of lymphoid follicles of the lymphatic system located in the small intestine) according to the results of the PV studies on chimpanzees [1], but it is still unclear where exactly CV replicate in the intestine since the results of PV studies are often applied to other EV. In this line, Bopegamage et al. showed that viral RNA and VP1 protein were detected in the follicle-associated epithelium and the intestinal villi of orally infected outbred mice with CVB4 JVB, and CVB3 Nancy. However, CVB markers (viral RNA, VP1, and eGFP) were absent in the germinating centers (GCs) of the PPs, although IFN-α production was detected in these GCs of PPs and in the mucosal layer of the small intestines from infected mice [2]. The persistence of EV in the intestinal mucosa has been associated with the development of islet autoimmunity and T1D [3]. CVB persistence and dissemination to target organs is due to a successful evasion from the mucosal immune system of the gastrointestinal tract. Stone et al. showed that CVB3 could evade the immune response at the primary site of infection by preventing type I and III IFN production in human intestinal epithelial cells (IECs) through alteration of the expression of the signaling proteins MAVS (mitochondrial antiviral signaling protein) and TRIF (TIR-domain-containing adaptor-inducing interferon-β) and eIF4G (eukaryotic initiation factor 4G), which is a key factor in the protein translation [4].

It has been suggested that the onset of islet autoimmunity and T1D results from impaired central tolerance to β-cell antigens following CVB infection of the thymus, particularly during fetal and neonatal life. Indeed, CVB4 can infect and persist in the thymus of humans and mice, especially in thymic epithelial cells (TECs), resulting in the alteration of *Igf2* (Insulin-like growth factor 2) expression (a major self-peptide of the insulin family found in the thymus) and disruption of negative selection of autoreactive T cells [5]. Furthermore, in utero infection with CVB4 induces a decrease in the level of Igf2 and Myo7 (myosin 7, an autoantigen of heart) mRNAs and proteins in mouse thymus and a decrease in transcription factors involved in thymic-negative selection such as *Aire* (autoimmune regulator) and *Fezf2* (forebrain expressed zinc finger 2) in enriched mouse TECs [6]. It is well known that lifestyle and risk factors such as diabetes and hypertension play a major role in chronic kidney disease outcomes, but the involvement of CV infections in kidney injury has also been suggested in a NOD mouse model infected with CVB4 [7].

Various preventive and therapeutic strategies to combat EV infection, especially CVB, have been reported. Antiviral drugs against CVB targeting viral or host proteins and formalin-inactivated or virus-like particles vaccines have been developed [3]. Interferon immunotherapy could be an alternative strategy since type I IFNs have been shown to confer strong protection against CVB replication. In this regard, Stone et al. demonstrated that type I (IFN-α) and type III IFNs (IFN-λ1, IFN-λ2) treatment can induce upregulation of several interferon-stimulated genes and can significantly reduce CVB3 replication in IECs and thus could be beneficial in the prevention of acute or persistent CVB infection in the gastrointestinal tract [4]. A vaccine derived from an attenuated strain of CVB3 with a mutation in the coxsackievirus–adenovirus receptor-interacting region (Mt10 vaccine) has been shown to induce cross-reactive neutralizing antibodies against CVB1, CVB3, and CVB4 and to protect against wild-type CVB3-induced myocarditis and pancreatitis in A/J mice [8].

In summary, the complex virus–host interactions have been addressed in original articles and reviews published in the Special Issue “Coxsackievirus Infection and Associated Diseases”, and it provides readers with a better understanding of some aspects of this topic. Further investigation could benefit the research of innovative therapeutic strategies to combat CV infections and associated diseases.
